# *TP53* codon 72 Gene Polymorphism Paradox in Associated with Various Carcinoma Incidences, Invasiveness and Chemotherapy Responses

**Published:** 2008-12

**Authors:** Hung-Yu Lin, Chun-Hsiung Huang, Wen-Jen Wu, Li-Ching Chang, For-Wey Lung

**Affiliations:** 1 *Division of Urology, Department of Surgery, E-DA Hospital/I-SHOU University, Taiwan, ROC;*; 2 *Graduate Institute of Medicine, College of Medicine, Kaohsiung Medical University, Taiwan, ROC;*; 3 *Department of Urology, Kaohsiung Medical University, Taiwan, ROC;*; 4 *Department of Occupation Therapy, I-SHOU University, Taiwan, ROC;*; 5 *Department of Medicine, Kaohsiung Armed Forces General Hospital, Taiwan, ROC;*; 6 *Graduate Institute of Behavioral Sciences, Kaohsiung Medical University, Taiwan, ROC;*; 7 *Department of Psychiatry, National Defense Medical Center, Taiwan, ROC;*; 8 *Calo Psychiatric Center, Taiwan, ROC*

**Keywords:** *TP53*, codon 72, apoptosis

## Abstract

*TP53* is the most common mutated gene in human cancers. Approximately half of all human malignancies exhibit *TP53* mutations. The *TP53* codon 72 polymorphism is a single-nucleotide polymorphism (SNP) in exon 4, resulting in the expression of either arginine (CGC) or proline (CCC) residues. In this article, we review literatures published in MEDLINE, and attempt to describe how these two polymorphic variants of *TP53* are functionally distinct, and how they influence cancer vulnerability and response to chemotherapy. The Arg72 variant has been shown to be more likely to induce apoptosis than the Pro72 variant, due to its ability to localize itself to mitochondria and trigger the release of cytochrome c into the cytosol. However, the influence of the *TP53* codon 72 polymorphism on the risk of developing various cancers, and their progression remains inconclusive because there has been no sustained evidence supporting a crucial role for the codon 72 variant in cancer therapy till now. We hypothesize that *TP53* gene might not only be involved in cell cycle control and the apoptosis induction response to DNA damage, but may also modulate individual cancer risk, and that this may correlate with the biofunctions of the two codon 72 variants. Additionally, latent factors might function synergistically with codon 72 variants to confer susceptibility to cancer development, progression, prognosis, and therapeutic responsiveness. Further etiological investigations are essential to reveal the association of and interaction between genetic and environmental factors in relation to carcinogenesis.

## INTRODUCTION

Oncogenic activity or loss of tumor suppressor function results from genetic alterations, such as mutation, insertion, or deletion within tumor cells, which ultimately causes abnormal gene expression (1). The *TP53* gene is a well-characterized tumor suppressor gene, and the most frequently mutated gene in various human cancer. *p53* protein also serves as a DNA motif binding-specific transcription factor in the regulation of genes that promote growth arrest or apoptotic response to cellular stress (2). The role of the *TP53* gene as the “guardian of the genome” has been studied in great detail; especially, it is able to successfully regulate *TP21* expression, a cell-cycle control gene, in response to DNA damage. Half of all human malignancies, including colon, bone, lung, breast, lymphoma, cervical, skin, gastric, ovary, brain and urological cancers, exhibit *TP53* mutations, either in a single hotspot or at multiple sites, frequently accompanied by wild-type *TP53* inactivation.

According to mutational analysis, *TP53* mutations most frequently occur within a highly conserved region comprising exons 4-8 (3). Many studies have focused on investigating whether *TP53* exon 4-8 mutations are associated with various cancers in tumor grade, stage, metastasis, tumor progression, tumor prognosis and response to different anticancer drugs (4-7). Unfortunately, the results have not been consistent.

Approximately 20 years ago, a new *TP53* mutation structure was discovered based on mobility differences when performing *p53* protein electrophoresis, resulting in a new concept of SNP and carcinogenesis. The mobility difference was due to a polymorphism located at *TP53* codon 72 (8). The *TP53* codon 72 polymorphism is a proline to arginine substitution which originally was thought of as a normal genetic variation without additional association with carcinogenesis. However, this concept was gradually disproved, after more advanced evidence of the association of the codon 72 polymorphism with cancer development had accumulated.

According to a previous study, the Arg72 variant was characterized by its increased ability to induce aptosis the Pro72 variant was suggested to more efficiently trigger G1 growth arrest. Additionally, several studies also indicated that the Pro72 variant more efficiently activated *TP53*-dependent DNA repair-related genes in different cell-based assays. Moreover, the Pro72 variant-bearing cells also exhibited reduced micronuclei formation, suggesting that it promoted greater genomic stability. Therefore, these two genetic variants were thought to function distinctly from one another, and their expression levels also modulated cancer risk (9) due to the uncovered associations with cancer progression, metastasis and anticancer drug treatment responses. However, whether the polymorphism plays an important role or functions dominantly with cancer development is still uncertain and paradoxical.

## *TP53* CODON 72 POLYMORPHISM

A SNP in exon 4 results in expression of either arginine (72R) or proline (72P) at codon 72 of *TP53*. This polymorphism is located in a proline-rich domain, which is essential for its DNA-binding ability to induce apoptosis. The two genetic variants (Pro 72 and Arg 72) had been thought of as having different functional activities. Marin *et al*. reported that some tumor-derived *TP53* mutants can bind to *TP73* and inactivate its biofunction. Moreover, this binding activity of *TP53* mutants can be interrupted by whether the *TP53* variants are encoded by codon 72, a common polymorphism in the human population. Generally, Arg72 might retain a less prohibitory effect to avoiding mutant *TP53* binding to *TP73*. The *TP73* gene, a *TP53* homologue, can activate *TP53*-responsive promoters and induce apoptosis in *TP53*-deficted cells. The ability of mutant *TP53* to bind *TP73*, neutralize *TP73*-induced apoptosis and transform cells in cooperation with EJ-Ras was enhanced when codon 72 encoded Arg (10). The Arg72 variant also appeared to more effectively interact with HPV-E6 *in vitro*, and to more easily be degraded through the ubiquitin proteasome pathway, resulting in inactivation of *TP53* gene and the induction of HPV-related tumor development (11). Thereafter, Thomas *et al*. revealed that Arg72 more likely acts dominantly in transcriptional regulation of *TP53* downstream targets that induce apoptosis or repress the transformation of primary cells (12).

In 1999, it was shown that the two genetic variants of *TP53* codon 72 were not functionally equivalent and the Arg72 variant induces apoptosis and suppresses tumor growth more efficiently than the Pro72 variant. Another study provided further evidence that the Arg72 variant is at least 5 times more efficient in apoptosis induction, by comparing it with the Pro72 variant (13). Furthermore, the Pro72 variant appears to induce higher levels of G1 arrest (14).

Siddique *et al*. suggested *TP53* is arguably the most critical tumor suppressor for inhibiting malignant transformation (9). Other than gain-of-function mutations, its genetic polymorphisms have been suggested to be risk factors for carcinogenesis. The Pro72 variant is crucial for specifically activating the *TP53*-dependent DNA repair genes in different cells, resulting in higher DNA-repair efficiency *in vitro*. Furthermore, Pro72 variant-expressed cells exhibit reduced micronuclei formation, compared with Arg72, suggesting that genomic instability is reduced in these cells. Taken together, these data provide further evidence that *TP53* codon 72 might function differently and somehow confer vulnerability to cancer (9). Interestingly, another study reported that fibroblasts and lymphocytes isolated from Arg72 homozygote centenarians and sexagenarians (Arg+) underwent oxidative-stress-induced apoptosis with higher proportions than those of proline allele carriers (Pro+). These data suggest that the *TP53* codon 72 polymorphism might contribute to apoptotic variability among the elderly, which is potentially relevant to age-related pathologic conditions, such as myocardial ischemia (15).

It was of interest that the Arg72 variant retained a higher apoptosis induction capability. One mechanism is that the Arg72 form may have a higher potential to localize itself to mitochondria, which might consequently trigger *p53*-mediated apoptosis. Mitochondrial localization of *TP53* gene is known to contribute to *TP53*-dependent apoptosis, as *TP53* mutants with compromised ability to localize to the mitochondria are also impaired in inducing apoptosis (16). Thus, the ability to localize to mitochondria might vary in different cell types and correspond to different cellular activities of mutant *TP53* and Arg72 variants. This has not yet been clarified and awaits further investigation. In fact, codon 72 polymorphism is essential for the full activation of *p53*-mediated apoptosis, since its location is within a proline-rich domain. We further tested two genetic variants in their apoptosis induction capability via inducible constructs to ectopic-expressed codon 72 variants in cells with endogenous *TP53* gene. The results indicate that pharmorubicin works better in cytotoxicity against bladder cancer Cell Line T24 (CGC, CGC) than bladder cancer Cell Line 5637 (CCC, CCC). For the codon 72 single nucleotide polymorphism vs. cell cycle, flow cytometry was used to analyze the formation of sub-GI, the apoptosis indicator for the cell cycle and cell procedural death of T24, T24 (C), T24 (G), and 5637, 56374 (C) and 5637 (G) in 0, 3, 6, 9, 12, 24 hours after Pharmorubicin acts. Nine and 12 hours after Pharmorubicin acts, Cell T24 has a higher ratio of sub-G1 than T24 (C) and T24 (G). As expected, the Arg72 variant induces apoptosis markedly better than the Pro72 variant. Our preliminary data also replicated the findings of Arg72 triggering apoptosis more effectively, due to its localizing to mitochondria which accompany cytochrome c release into cytosol, one of the hallmarks of apoptosis (9, 13). In addition, the Arg72 variant might also interact with *MDM2*, leading to enhanced nuclear export in the ubiquitin-mediated protein degradation process. This result was successfully confirmed by immuncytochemical staining of cell lines that inducibly expressed the Arg72 variant (13).

These results support previous findings that *p53*-mediated apoptosis might be due to the ability of the Arg72 variant to localize to mitochondria. In addition, the nuclear export rate of *TP53* gene might also influence the mitochondrial localization of *P53*. Thus, the genetic polymorphism might correspond to individual differences of apoptosis induction, and the *TP53* codon 72 polymorphism might be the major determinant in this process. The current hypotheses that attempt to explain why Arg72 has a higher *p53*-mediated apoptosis induction capability can be divided into two major categories. First, the Arg72 variant retains a higher potential to localize itself to mitochondria; hence, this cellular activity might provide an opportunity to enhance interaction between *TP53* and *MDM2*. *MDM2* is an ubiquitin ligase (E3) that binds to *TP53* gene and triggers its ubiquitination toward nuclear exportation, which accompanies cytochrome c release. Second, the Arg72 variant was found to interact with the pro-apoptotic gene product “PREP” more intensively, when compared to the Pro72 variant. This interaction would result in enhancing apoptosis activity more than 10-fold. To sum up, these studies fully illustrate that the *TP53* codon 72 polymorphism leads to amino acid changes, and its variant also acts differently; however, more precise functional studies of codon 72 variants should be carried out in order to uncover the in-depth molecular mechanism that is associated with different illnesses.

## THE RELATIONSHIP BETWEEN THE *P53* CODON72 POLYMORPHISM AND VARIOUS CLINICAL CANCERS

Human genetic polymorphisms have recently been associated with the development of various cancers and with therapeutic responses to anticancer drugs. Sullivan *et al*. demonstrated that cellular responses to anticancer agents were strongly influenced by different *TP53* SNP sites *in vitro*. Functionally expressed Pro72 variants (wild-type *TP53* gene), through an inducible vector system in the cells with endogenous wild-type *TP53* gene, predominantly resulted in G1 growth arrest; only a small number of cells were observed to undergo apoptosis at drug concentrations causing extensive apoptosis in cells expressing the Arg72 variant (17). Cortezzi *et al*. evaluated the role of HPV infection and the *TP53* polymorphism in head and neck cancers by analyzing 50 tumors, as well as 142 swabs of oral mucosa from control individuals. The frequency distribution of genotypes in the controls was 50% Arg/Arg, 43% Arg/Pro and 7% Pro/Pro; whereas in tumors, it was 52% Arg/Arg, 32% Arg/Pro, and 16% Pro/Pro. The Arginine residue was a major allele that appeared more frequently in the controls, indicating it might serve as a protective factor against head and neck cancers. The data also showed that HPV infection did not result in an increased risk of head and neck tumor development (18).

Yang investigated *TP53* codon 72 polymorphisms in 435 patients with esophageal squamous cell carcinoma (ESCC) and 550 cancer-free subjects from the same geographical region (19). The *TP53* Arg/Arg genotype was significantly increased in ESCC cases compared with the control subjects (85.7 vs. 49.6%, *P*<0.001), resulting in an elevated ESCC risk (OR = 6.48, 95% CI = 4.65-9.03). *TP53* Arg/Arg homozygosity, HPV infection, smoking, and alcohol using may synergistically increase the risk of ESCC development (19).

However, results of studies of codon 72 polymorphism association with cancer have been inconsistent. Sousa *et al*. performed a meta-analysis by reviewing studies using sample sets of a European population origin to summarize the global risk of this polymorphism covariate with geographical/ethnic differences in defining the genetic profile of each individual and their susceptibility to cervical cancer development (20). Unexpectedly, the results indicated that *TP53* Arg/Arg homozygosity did not represent a predisposing factor predicting the development of cervical lesions in the majority of the European countries studied. However, in countries with low incidence rates of cervical cancer, this polymorphism might potentially be used as a biomarker for disease diagnosis (20).

The interaction of HPV-E6 with *TP53* gene was suggested as the most important cellular event resulting in HPV-associated carcinogenesis, but few reports have corroborated this finding; there was even a lack of information on the role of the *TP53* codon 72 polymorphism in the development of HPV-E6-associated cancers. Although no association between the *TP53* codon 72 polymorphism and HPV-E6-related cancers was found, some reports stated that Arg72 homozygosity was associated with the carcinogenesis of breast cancer (21, 22) and bladder cancers (23). Brant *et al*. genotyped a group of 77 patients with advanced HNSCC, using direct sequencing, and a significantly higher frequency of homozygosity of Arg72 was detected among the subjects (24). Unfortunately, the impact of this polymorphism on the disease risk of squamous cell carcinoma of the head and neck (HNSCC) has not yet been established and remains unclear due to contradictory results.

Regarding Pro72 homozygosity, Wang *et al*. investigated the *TP53* codon 72 polymorphism in 194 lung cancer patients and 152 noncancerous controls in a Taiwanese population. It was suggested that the Pro72 variant might represent a risk allele associated with an increased risk of lung cancer among female subjects. Moreover, the *TP53* codon 72 polymorphism might also play a role in cancer susceptibility and prognosis in a specific subgroup of lung cancer patients in Taiwan (25). An association between the Pro/Pro genotype of the *TP53* codon 72 polymorphism and lung cancer has been reported previously (26). Hu *et al*. further examined the effect of this polymorphism and other *TP53* mutations on the survival of non-small cell lung cancer (NSCLC) victims (27). Paired samples were collected from 182 patients with NSCLC. *TP53* mutations were successfully detected in 93 of 182 (51%) tumors by direct sequencing and/or the Gene Chip *TP53* assay, and the *TP53* codon72 polymorphism was identified by PCR-RFLP. It was shown that the *TP53* Pro72 allele was associated with an increasing frequency of *TP53* mutations in NSCLC (27), but no association was found for this polymorphism with lung cancer disease risk or prognosis.

In 2003, Kuroda *et al*. investigated the *TP53* codon 72 polymorphism in 112 male urothelial cancer cases and 175 male unrelated non-cancer controls (28). It was suggested that the Pro/Pro genotype might be associated with an increased risk of urothelial cancer among smokers (28). Bergamaschi *et al*. found that the Pro72 allele occurred more frequently in patients with chronic myeloid leukemia (CML) than in controls, and among CML patients who had no cytogenetic response rather than among responders (29). In addition, Zhu *et al*. examined the association between the *TP53* codon 72 polymorphism and colorectal cancer risk in 345 patients with colorectal cancer and 670 controls in a Chinese population (30). They concluded that the *p53* codon 72 polymorphism may contribute to the etiology of colorectal cancer in a Chinese population, particularly among alcohol consumers (30).

Ezzikouri *et al*. evaluated the association between the *TP53* codon 72 polymorphism and hepatocellular carcinoma (HCC) in a Moroccan population (31). The results showed that the patients with HCC had a higher frequency of Pro72 homozygosity (13.5% vs. 6.3%, *P*<0.02) compared with controls, and resulted in a 2.3-fold increased risk of liver cancer development (odds ratio [OR], 2.304; 95% confidence interval [CI], 1.014-5.234) (31).

Hadhri-Guiga *et al*. utilized PCR-RFLP to detect the *TP53* codon 72 polymorphism in peripheral blood samples from 115 patients with nasopharyngeal carcinoma (NPC) and 83 healthy individuals (32). The results provided evidence that individuals with the Pro/Pro genotype had an increased risk of developing NPC in Tunisia (32).

In reviewing the SNP-association studies of the *TP53* codon 72 polymorphism, it was hard to define whether the genotype or the allele carriage was actually associated with cancer vulnerability; in addition, it seems natural that gene associations might vary with different samples, methodologies, cancer types, and ethnic heterogeneity. Indeed, functional abnormality of *TP53* is commonly associated with various cancers. High-grade and late-stage bladder cancers have been reported to have high levels of expression of *TP53* mutant genes or that the *TP53* gene inactivation, as identified by immunohistochemical staining. More recently, the *PT53* codon 72 polymorphism was extensively studied to determine cancer risk, and both Arg72 and Pro72 variants were considered as risk factors for the development of various cancers.

This polymorphism also revealed a potential use in predicting the therapeutic efficacy and responses of anti-cancer agents. Vikhanskaya evaluated the effect of six *TP53* hotspot mutations (R175H, G245S, R248W, R249S, R273H, and R282W), in conjunction with the codon 72 polymorphism, on various anticancer drugs, either alone or in combination in laboratory generated isogenic lung cancer cell lines (33). It was shown that the codon 72 polymorphism and *PT53* mutations could be integrated as biomarkers for the prediction of treatment response, although variables for each cancer type require further detailed evaluation (33). Yuan *et al*. studied the responsiveness of 165 patients with advanced non-small cell lung cancer to platinum-based chemotherapy with p53 and p73 polymorphisms and found the p53 Pro allele carriers had higher response rate than non-carriers (OR = 2.46; 95% CI = 1.11 - 5.45) (34).

Han *et al*. analyzed 148 advanced non-small cell lung cancer treated with different anticancer drug to investigate whether polymorphisms of *p53* codon 72 (Arg72Pro) and MDM2 SNP309 (309T>G) affect *p53* expression and the clinical outcome of patients (35). Genotypes were correlated with *p53* expression, clinicopathologic factors, tumor response, and survival. The results indicate that patients with the Pro/Pro variant were more likely to be resistant to first-line chemotherapy, especially to irinotecan plus cisplatin regimen, than those with Arg/Arg or Arg/Pro variants (60% vs 27%, *P*=0.014). In multivariate analysis, the Pro/Pro genotype was strongly predictive for shorter progression-free survival (35). Similarly results are also found in breast cancer who received adjuvant chemotherapy (36). Galic *et al*. investigated the relationship between codon 72 polymorphisms in *TP53* and clinical outcomes in 188 women with ovarian and peritoneal carcinomas (37). Then, they found that women with the codon 72 Pro/Pro had a decreased overall survival (median, 29 months) compared with women with one or two arginine alleles (median, 49 months; *P*=0.04) (37).

*PT53* codon 72 polymorphism has been connected with cancer invasiveness. Chen *et al*. indicated that Pro72 homozygosity was more frequently found in the invasive group than the non-invasive group (25% and 2.9%, respectively, *P*<0.001) (38). Additionally, more than 70% of the non-invasive bladder cancers were Arg72 homozygotes. This result was consistent with reports of hepatocellular carcinoma that showed an association of chronic liver disease history with Pro72 homozygosity, in a study by Yu *et al*. Pro72 homozygosity has been suggested to be associated with the tumor invasiveness of bladder cancer (38). The *TP53* codon 72 polymorphism also has been associated with tumor staging and cancer progression. Lung *et al*. indicated a significant relationship between the *PT53* codon 72 polymorphism and colorectal cancer in a Taiwanese population (39). Pro72 homozygosity was also shown to have a higher risk of association with an onset of cancerous disease; it was estimated that risk increased 1.70 times for each grade change (Dukes A-D), indicating an advanced association with cancer progression (39). Even though positive linkage of the *TP53* codon 72 polymorphism to cancerous phenotypes has been observed, negative findings have led to doubts about this association. To determine whether the variant influences the individual risk of urologic cancer and/or its progression, Wu *et al*. analyzed allelic frequencies of the *TP53* codon 72 polymorphism in 85 renal cell carcinoma patients, 151 urothelial cancer patients, 33 testicular cancer patients, 28 prostatic cancer patients and 56 patients without neoplastic disease, using PCR-RFLP; no association was found between three independent genotypes and tumor grade or staging (40). Idbaih *et al*. suggested that there was no association between oligodendroglial tumors and the *PT53* codon 72 polymorphism. Similarly, no correlation was found for disease prognosis, *PT53* expression level, and chromosomes 1p and 19q status (41). Similarly, Phang *et al*. suggested that *p53* codon 72 polymorphism was not associated with cancer predisposition, cancer risk, onset age, overall survival and response to therapy in Chinese leukaemia patients (42). Thus, Siddique concluded that the findings on whether a genetic variant of the *PT53* codon 72 polymorphism might be significantly associated with carcinogenesis were inconsistent (43). It was proposed that genetic variants of *PT53* codon 72 might be ethnicity-specific, selectively regulated distinct signaling pathways, with which the Arg allele was activated during cancer development in Asians. Hence, the expression level of each variant, rather than the genotypic status, might be a useful indicator for risk assessment of cancer susceptibility (43).

## CONCLUSION

The *PT53* codon 72 polymorphism has been widely studied and discussed in terms of cancer development and prognosis in clinics. Whether the variant (Arg72 or Pro72) is important or dominantly associated with cancer development or prognosis is still uncertain and paradoxical.

The *PT53*-Arg72 and *PT53*-Pro72 variants play important roles in influencing the chemotherapeutic response of various cancers *in vitro* and *in vivo*. However, findings regarding whether expression of either variant is important to cancer therapy are inconclusive.

The germline *PT53* codon 72 polymorphism does alter its biological functions and might confer individual susceptibility to various cancers, but the influences of these two variants on the development of various cancers, and their progression, prognosis and response to therapy may be partially cancer type-specific. The roles of the two genetic variants have been identified, but uncovering the underlying molecular mechanisms awaits further investigation. Other than susceptible SNPs, gene-gene interaction and the interplay and relationship of genetic components and environmental factors are thought to important roles in modulating cancer vulnerability.
